# Recent advances in the compound-oriented and pattern-oriented approaches to the quality control of herbal medicines

**DOI:** 10.1186/1749-8546-3-9

**Published:** 2008-08-04

**Authors:** Zhongda Zeng, Foo-tim Chau, Hoi-yan Chan, Chui-yee Cheung, Tsui-yan Lau, Shuiyin Wei, Daniel Kam-wah Mok, Chi-on Chan, Yizeng Liang

**Affiliations:** 1 Chemometrics and Herbal Medicine Laboratory, Department of Applied Biology and Chemical Technology, Hong Kong Polytechnic University, Hong Kong SAR, PR China; 2 Research Centre of Modernization of Chinese Medicine, College of Chemistry and Chemical Engineering, Central South University, Changsha 410083, PR China; 3 State Key Laboratory of Chinese Medicine and Molecular Pharmacology, Shenzhen 518057, PR China

## Abstract

The current approaches to the quality control of herbal medicines are either compound-oriented or pattern-oriented, the former targeting specific components with some known chemical properties and the latter targeting all detectable components. The marker approach uses specific chemical compounds with known molecular structures, while the multi-compound approach uses both chemical compounds with known structures and those with partial chemical information e.g. retention times, mass spectra and ultraviolet spectra. Apart from chromatographic techniques, new techniques such as oscillating and electrochemistry fingerprints have been developed for quality control. Chemometric resolution methods are widely used for component deconvolution and data comparison. Pattern recognition techniques are used for authentication of herbal medicines.

## Background

High chemical complexity of herbal medicines makes quality control through chemical analysis difficult. For example, a common Chinese medicinal herb *Cortex Cinnamomi *(*Rougui*) revealed over 90 volatile components in a gas chromatography-mass spectroscopy (GC-MS) experiment, only 60 of which were chemically identified [1]. Little chemical information is known about herbal medicines [2]. Mok and Chau categorized authentication and quality control of herbal medicines into the 'component-based' and 'pattern-based' approaches [3]. For a refined classification, we refer to these two approaches in this article as the compound-oriented approach and pattern-oriented approach respectively. The compound-oriented approach includes the marker approach and the multi-compound approach. The marker approach takes into account herbal medicines with known components, while the multi-compound approach may include some unknown components whose chemical properties are known. The pattern-oriented approach, such as fingerprint analysis, evaluates all data acquired from analytical instrument. For example, fingerprints and bioactivities have recently been correlated to the quality control of herbal medicines [4-6]. Instead of measuring all elements within a biological system, systems biology aims to reveal intrinsic trends of the system [7,8]. Metabonomics focuses on the investigation of high-throughput data from metabolite profiling. The key advantage of metabonomics is to provide an integrative and systematic view of metabolism, which may also reveal the quality of herbal medicines [9,10].

Different ingredients within a herbal medicine may have synergistic effects. These active ingredients must be identified and quantified for better understanding of the action mechanisms of herbal medicines. For the moment, the conventional marker approach is not successful as only a few chemical markers are available for herbal medicines in the American Herbal Pharmacopeia and Chinese Pharmacopoeia. For example, in the Chinese Pharmacopoeia (2005 edition) only one chemical marker chlorogenic acid (C_16_H_18_O_9_) is recommended for the identification of *Flos Lonicerae *(*Jinyinhua*) (content ≥ 1.5%) and *Flos Chrysanthemi *(*Juhua*) (content ≥ 0.2%) [11]. These two herbs cannot be differentiated with the only chemical marker. Likewise, *Fufang Danshen Diwan *is a formulated herbal medicine that consists of three herbs but there is only one chemical marker salvianic acid A (C_9_H_10_O_5_) recommended for this particular medicine in the Chinese Pharmacopoeia (2005 edition) [11].

The pattern-oriented approach such as fingerprinting is more useful than compound-oriented approach in most cases. Chemical fingerprints are characteristic for herbal medicines, and are therefore useful in the quality control of herbal medicines [12-15]. The information-rich chemical fingerprints can be obtained from advanced instruments [16-21]. All detectable chemical components of a herbal medicine may be shown in fingerprints. Chemometrics helps analyze and interpret useful information from raw data, e.g. alignment of shifts of retention time in chromatography, data assessment and comparison, smoothing and filtering, deconvolution and resolution of overlapping peak clusters, in determination of the chromatographic and spectral profiles of pure components [22-27]. This article summarizes recent advances in both compound-oriented and pattern-oriented approaches in terms of experimentation, instrumentation and data processing.

### Compound-oriented approach

#### Marker approach

According to the Chinese Pharmacopoeia (2005 edition), identification and quantification of chemical markers are crucial to the quality control of herbal medicines. A total of 525 quantitative assays of chemical markers were documented in the Chinese Pharmacopoeia (2005 edition) for assessment of herbal medicinal materials, plant lipids, herbal extracts and formulations [11]. Chemistry of these markers is known and their analytical procedures and reference standards are available for quality control. Chau *et al*. used near infrared spectroscopy (NIR) to quantitatively determine the content of berberine and total alkaloid in *Cortex Phellodendri *(*Guanhuangbo*) [28]. The content of berberine determined by high-performance liquid chromatography-diode array detection (HPLC-DAD) was used as a critical parameter to confirm the accuracy of the data obtained from NIR according to a linear model of partial least squares (PLS) regression. In another study, high-performance liquid chromatography (HPLC) and HPLC-DAD were used to assess the quality consistency of a formulated Chinese medicine *Qingfu Guanjie Shu *(capsule) using four marker compounds, namely sinomenine, paeoniflorin, paeonol and curcumin. [29]. Lin *et al*. used liquid chromatography-tandem mass spectrometers (LC-MS/MS), solid phase extraction, and the marker glycyrrhetic acid to simultaneously validate *Radix Glycyrrhizae *(*Gancao*) and quantify the target compound in the samples [30]. Quantitative studies of markers and identification of active ingredients were carried out for the quality control of herbal medicines [31-34]. Gas chromatography (GC), gas chromatography-mass spectroscopy (GC-MS), thin layer chromatography (TLC), thin layer chromatography-ultraviolet spectrophotometry (TLC-UV), capillary electrophoresis (CE) and capillary zone electrophoresis (CZE) were also proposed for the quality control of herbal medicines [35-39].

#### Multi-compound approach

Compared with the marker approach, the multi-compound approach uses multiple compounds with known chemical properties and does not necessarily require chemical markers. Chemometric deconvolution and resolution are major methods in this approach. In the Chinese Pharmacopoeia (2005 edition) [11], multiple compounds, instead of a single compound, are recommended for the quality control of herbal medicines. For example, total flavonol glycosides (i.e. quercetin, isorhamnetin and kaempferol) as well as total terpene lactones (i.e. bilobalide, and ginkgolides A, B and C) were used for the quality control of a ginkgo leaf product [11]. However, analyzing multiple compounds in a single chromatogram may not be easy. These chromatograms often contain overlapping peaks, which may not be resolved by changing chromatographic conditions. One possible solution is the use of chemical and/or instrumental methods that take advantage of spectra with very close retention times, e.g. mass spectra, ultraviolet spectra or other chemical properties containing variations large enough to resolve overlapping chromatographic profiles [40-43].

Chemometric resolution methods (CRM) were used extensively in the past decades to 'purify' chromatographic peak profiles of complex mixture systems such as herbal medicines [13]. The qualitative and quantitative chemical information obtained by CRM did help discover the active ingredients of herbal medicines and study the synergistic effects of the ingredients [1].

Previously, both iterative and non-iterative resolution methods were used to study the volatile and non-volatile components in herbal medicines [44-46]. Many non-iterative resolution methods such as heuristic evolving latent projection (HELP), alternative moving window factor analysis (AMWFA), (subwindow factor analysis) SFA, evolving window orthogonal projection (EWOP) were useful in discovering more than ten components of herbal medicines [47-51]. Using GC-MS coupled with HELP, Li *et al*. identified 38 volatile chemical components of *Radix Paeoniae Rubra *(*Chishao*), which accounted for 95.21% of all detectable components [52]. In another study, 69 components of *Radix Rehmanniae Preparata *(*Shudihuang*) were separated, of which 59 were identified using standard spectra in the database of the National Institute of Standards and Technology (NIST). The 26 identified methyl esters accounted for about 94.29% of the total number of components [53]. Most of the iterative methods including (orthogonal projection approach) OPA and (iterative orthogonal projection resolution) IOP were applied to determine the chemical composition of herbal medicines [45,46]. With these chemometric methods, 65 volatile chemical constituents of *Rhizoma et Radix Notopterygii *(*Qianghuo*) were identified out of the 98 separated chemicals. Qi *et al*. resolved the overlapping chromatographic peaks in *Resina Draconis *(*Xuejie*) using HPLC-DAD. Therefore, using chemometric methods with hyphenated instruments was powerful in the analysis of herbal medicines [54]. Zeng *et al*. used PCA and generalized rank annihilation factor analysis (GRAFA) to process HPLC-DAD data sets obtained from *Radix Salviae Miltiorrhizae *(*Danshen*) and *Radix Notoginseng *(*Sanqi*) [55]. Ye *et al*. simultaneously analyzed seven major saponins of *Danshen Diwan *with HPLC-DAD and electron spray ionization-mass spectrometry (ESI-MS) [56].

Chemometric methods including spectral correlative chromatography (SCC), multi-component spectral correlative chromatography (MSCC), AMWFA were proposed for comparing three-dimensional (3D) or two-dimensional (2D) chromatographic profiles and integrating presence or absence information [49,57-59]. SCC was used to compare pure or selective herbal medicine components [59]. Both MSCC and AMWFA were used to analyze complex herbal medicines. The main feature of MSCC is the construction of an orthogonal projection matrix using abstract spectra acquired from decomposition of original fingerprint data sets. For comparison, the pure and mixed spectra are projected to the matrix for presence or absence information of target components. AMWFA was used to extract pure chromatographic and spectral profiles of common components of related herbal medicines [49,58,59]. All these new chemometric algorithms were applied in the identification, quantification, comparison of chemical components and quality control of herbal medicines [60-66].

### Pattern-oriented approach

#### Single pattern approach

##### Fingerprint analysis

The pattern-oriented approach analyzes fingerprints obtained from one-, two- or higher dimensional chromatographic and/or spectral instruments. Single pattern approach focuses on one type of pattern (e.g. chemical fingerprints of chromatograms and spectra) for the quality control of herbal medicines. Significant progress was made in 2D chromatographic profiles obtained from HPLC, CE, GC as well as 3D ones from hyphenated instruments [18,19,67-70]. Yan *et al*. combined 3D fingerprints from HPLC-DAD instruments with principal component analysis (PCA) to monitor *Qingkailing *(a proprietary Chinese medicine injection formulation) produced by various manufacturers [71]. High-speed counter-current chromatography (HSCCC) and high-performance liquid chromatography-coulometric electrode array detector (HPLC-CEAD) were used to obtain the fingerprint of *Radix Salviae Miltiorrhizae *(*Danshen*) collected from various localities [72-74]. Binary chromatographic fingerprints from HPLC-DAD and GC-MS were used to analyze the aporphinoid and quinolizidine alkaloids of *Caulophyllum robustum *(*Leiyemudan*). Similarity index and the cluster analysis method were used to analyze the quality of ten batches of samples of *Caulophyllum robustum *[75]. Fan *et al*. developed multiple chromatographic fingerprinting including two HPLC fingerprints for the quality assessment of *Danshen Diwan *[76]. Thin layer chromatography scan (TLCS) provided unique fingerprints which differentiated passiflora and other herbs grown under various conditions [21,77]. Fourier transform infrared spectroscopy (FT-IR), NIR and two-dimensional correlation infrared spectroscopy (2D-IR) spectroscopy were used to construct spectral fingerprints of complex herbal medicines [20,28,78,79]. New fingerprinting techniques have recently emerged, such as oscillating fingerprints [80], electrochemistry fingerprints [81,82], X-ray diffraction (XRD) second derivative fingerprints [83] and high performance liquid chromatography electrospray ionization tandem mass spectrometry (HPLC-ESI-MSn) [84].

##### Chemometric methods for fingerprint analysis

It is important to extract useful information from chromatographic/spectral fingerprints which contain chemical information of all detectable components of herbal medicines. Chau *et al*. developed a software package Computer Aided Similarity Evaluation (CASE) for data processing under Matlab [85]. Gong *et al*. developed a series of chemometric methods to extract information from fingerprints [24,86,87]. Xu *et al*. used target peak alignment (TPA) to correct the retention time shifts and multiplicative scattering correction (MSC) for response correction [88]. In addition, wavelet technique was used for data pre-processing of fingerprints [89]. Comparison, assessment and discrimination analysis are key steps to process the 'standardized' fingerprints of herbal medicines. Chemometric methods are used to compare data among herbs with hundreds or even thousands of chemical components. This information can be further used to identify the origins of herbs, or to authenticate herbs. For example, research has made significant progress since the introduction of the similarity index and pattern recognition analysis [90-94]. Information fusion and determination of relative entropy critical value were proposed for the similarity analysis of fingerprints of herbal medicines [90-93]. PCA, orthogonal projection technique (OP), 2D-IR worked well on data discrimination analysis of fingerprints [94-97]. Data deconvolution methods helped obtain chromatographic and spectral profiles of individual pure components [47-53]. Furthermore, chemical fingerprint databases of herbal medicines were developed according to chemometric methods [98,99].

#### Multi-pattern approach

The multi-pattern approach assesses the quality of herbal medicines according to multiple patterns. For example, both chromatographic fingerprints and biological activity profiles are used for the quality control of herbal medicines. This approach targets the discovery of bioactive ingredients, assessment of medical effects, correlation between chemical fingerprints and pharmacological indices, and quality control [100-105]. Some studies used chromatographic fingerprints from liquid chromatography-diode array detection-atmospheric pressure chemical ionization-mass spectroscopy (LC-DAD-APCI-MS) followed by data processing. Wang *et al*. found 32 potential bioactive components of *Radix Angelicae Sinensis *(*Danggui*) in rabbit plasma and over ten new compounds [100]. Using a *Herba Houttuyniae *(*Yuxingcao*) injection, Lu *et al*. evaluated its anti-inflammatory effects and established the correlation between the chemical components of the injection and the bioactivities of the active ingredients [101,102]. Wang *et al*. devised a method which combined metabolic profiling and liquid chromatography-diode array detection-mass spectroscopy (LC/DAD-MS) with chemometrics to study *Danggui Buxue Tang*, a Chinese medicine formulation [103]. Aided by PCA, Yu *et al*. discovered major bioactive components of *Aquilegia oxysepala *(*Jianeloudoucai*) [104]. Evidently, the multi-pattern approach provides a reliable means for the quality control of herbal medicines.

## Conclusion

The compound-oriented and pattern-oriented approaches to the quality control of herbal medicines have been significantly improved in terms of analytical instruments, biological screening methods and chemometrics. Among all the advanced techniques, multi-pattern approach will have a great potential for further development.

## Abbreviations

2D-IR: two-dimensional correlation infrared spectroscopy; AMWFA: alternative moving window factor analysis; CE: capillary electrophoresis; CRM: chemometric resolution methods; CZE: capillary zone electrophoresis; DAD: diode array detection; ESI-MS: electron spray ionization-mass spectrometry; EWOP: evolving window orthogonal projection; FSMWEFA: fixed-size moving window evolving factor analysis; FT-IR: Fourier transform infrared spectroscopy; GC: gas chromatography; GC-MS: gas chromatography-mass spectroscopy; GRAFA: generalized rank annihilation factor analysis; HELP: heuristic evolving latent projection; HPLC: High-performance liquid chromatography; HPLC-CEAD: high-performance liquid chromatography-coulometric electrode array detector; HPLC-DAD: high-performance liquid chromatography-diode array detection; HPLC-ESI-MSn: high-performance liquid chromatography electrospray ionization tandem mass spectrometry; HSCCC: high-speed counter-current chromatography; IOP: iterative orthogonal projection resolution; LC-DAD-APCI-MS: liquid chromatography-diode array detection-atmospheric pressure chemical ionization-mass spectroscopy; LC/DAD-MS: liquid chromatography-diode array detection-mass spectroscopy; LC-MS: liquid chromatography-mass spectroscopy; LC-MS/MS: liquid chromatography-tandem mass spectrometers; MS: mass spectroscopy, MSCC: multi-component spectral correlative chromatography; NIR: near infrared spectroscopy, OP: orthogonal projection technique; OPA: orthogonal projection approach, PCA: principle component analysis; PLS: partial least squares; SCC: spectral correlative chromatography; SFA: subwindow factor analysis, TLC: thin layer chromatography; TLC-UV: thin layer chromatography-ultraviolet spectrophotometry; XRD: X-ray diffraction.

## Competing interests

The authors declare that they have no competing interests.

## Authors' contributions

ZZ and FC conceived the classification of approaches to quality control of herbal medicines. ZZ drafted the manuscript. FC supervised the project and revised the manuscript. HC and CYC provided references and helped revise the manuscript. TL and SW advised on the manuscript. DM, COC and YL were responsible for some studies in this work. All authors read and approved the final manuscript.

## References

[B1] Gong F, Liang YZ, Fung YS (2004). Analysis of volatile components from *Cortex cinnamomi *with hyphenated chromatography and chemometric resolution. J Pharmaceut Biomed Anal.

[B2] Liang YZ, Kvalheim OM, Manne R (1993). White, gray and black multicomponent systems – a classification of mixture problems and methods for their quantitative analysis. Chemom Intell Lab Syst.

[B3] Mok DKW, Chau FT (2006). Chemical information of Chinese medicines: A challenge to chemist. Chemom Intell Lab Syst.

[B4] Lu HM, Liang YZ, Yi LZ, Wu XJ (2006). Anti-inflammatory effect of *Houttuynia cordata *injection. J Ethnopharmacol.

[B5] Yi ZB, Yu Y, Liang YZ, Zeng B (2007). Short communication Evaluation of the antimicrobial mode of berberine by LC/ESI-MS combined with principal component analysis. J Pharmaceut Biomed.

[B6] Wang YL, Liang YZ, Chen BM, He YK, Li BY, Hu QN (2005). LC-DAD-APCI-MS-based screening and analysis of the absorption and metabolite components in plasma from a rabbit administered an oral solution of danggui. Anal Bioanal Chem.

[B7] Nurse P (2003). Systems biology: Understanding cells. Nature.

[B8] Cottingham K (2005). Systems Biology: A Boon for Analytical Chemists?. Anal Chem.

[B9] Wang Y, Tang H, Nicholson JK, Hylands PJ, Sampson J, Holmes E (2005). A Metabonomic Strategy for the Detection of the Metabolic Effects of Chamomile (Matricaria recutita L.) Ingestion. J Agric Food Chem.

[B10] Van Dorsten FA, Daykin CA, Mulder TPJ, Van Duynhoven JPM (2006). Metabonomics Approach To Determine Metabolic Differences between Green Tea and Black Tea Consumption. J Agric Food Chem.

[B11] The State Pharmacopoeia Commission of People's Republic of China (2005). Pharmacopoeia of the People's Republic of China.

[B12] Xie PS, Chen SB, Liang YZ, Wang XH, Tian RT, Upton R (2006). Chromatographic fingerprint analysis-a rational approach for quality assessment of traditional Chinese herbal medicine. J Chromatogr A.

[B13] Liang YZ, Xie PS, Chan K (2004). Quality control of herbal medicines. J Chromatogr B.

[B14] Drašar P, Moravcova J (2004). Recent advances in analysis of Chinese medical plants and traditional medicines. J Chromatogr B.

[B15] Xie PS (2007). Discussion of the present situations, development and problems of fingerprint of Chinese medicines. Zhongyaocai.

[B16] Niu F, Niu ZQ, Xie GB, Fang M, Zhang GE, Cui Z, Tu PF (2006). Development of an HPLC fingerprint for quality control of Radix Semiaquilegiae. Chromatographia.

[B17] Xie GX, Qiu MF, Zhao AH, Jia W (2006). Fingerprint analysis of Flos Carthami by pressurized CEC and LC. Chromatographia.

[B18] Sun Y, Guo T, Sui Y, Li FM (2003). Fingerprint analysis of *Flos Carthami *by capillary electrophoresis. J Chromatogr B.

[B19] Zhao LH, Huang CY, Shan Z, Xiang BR, Mei LH (2005). Fingerprint analysis of *Psoralea corylifolia *L. by HPLC and LC-MS. J Chromatogr B.

[B20] Woo YA, Kim HJ, Ze KR, Chung H (2005). Near-infrared (NIR) spectroscopy for the non-destructive and fast determination of geographical origin of Angelicae gigantis Radix. J Pharmaceut Biomed.

[B21] Birk CD, Provensi G, Gosmann G (2005). TLC fingerprint of flavonoids and saponins from passiflora species. J Liq Chromatogr R T.

[B22] Li BY, Hu Y, Liang YZ, Xie PS, Du YP (2004). Quality evaluation of fingerprints of herbal medicine with chromatographic data. Anal Chim Acta.

[B23] Yin XL, Fang KT, Liang YZ, Wong RNS, HA AWY (2005). Assessing phylogenetic relationships of *Lycium *samples using RAPD and entropy theory. Acta Pharmacol Sin.

[B24] Gong F, Liang YZ, Fung YS, Chau FT (2004). Correction of retention time shifts for chromatographic fingerprints of herbal medicines. J Chromatogr A.

[B25] Liu HX, Sun SQ, Lv GH, Liang XY (2006). Discrimination of extracted lipophilic constituents of Angelica with multi-steps infrared macro-fingerprint method. Vib Spectrosc.

[B26] Xu CJ, Liang YZ, Chau FT (2005). Identification of essential components of *Houttuynia cordata *by gas chromatography/mass spectrometry and the integrated chemometric approach. Talanta.

[B27] Cai M, Zhou Y, Gesang SL, Bianba C, Ding LS (2006). Chemical fingerprint analysis of rhizomes of *Gymnadenia conopsea *by HPLC-DAD-MS*n*. J Chromatogr B.

[B28] Chan CO, Chu CC, Mok DKW, Chau FT (2007). Analysis of berberine and total alkaloid content in Cortex Phellodendri by near infrared spectroscopy (NIRS) compared with high-performance liquid chromatography coupled with ultra-visible spectrometric detection. Anal Chim Acta.

[B29] Xie Y, Jiang ZH, Zhou H, Cai X, Wong Yuen-F, Liu ZQ, Bian ZX, Xu HX, Liu L (2007). Combinative method using HPLC quantitative and qualitative analyses for quality consistency assessment of a herbal medicinal preparation. J Pharmaceut Biomed.

[B30] Lin ZPJ, Qiu SX, Wufuer A, Shum L (2005). Simultaneous determination of glycyrrhizin, a marker component in radix Glycyrrhizae, and its major metabolite glycyrrhetic acid in human plasma by LC-MS/MS. J Chromatogr B.

[B31] Huang LF, Liang YZ, Guo FQ, Zhou ZF, Chen BM (2003). Short communication Simultaneous separation and determination of active components in Cordyceps sinensis and Cordyceps militarris by LC/ESI-MS. J Pharmaceut Biomed.

[B32] He K, Pauli GF, Zheng B, Wang HK, Bai NS, Peng TS, Roller M, Zheng QY (2006). Cimicifuga species identification by high performance liquid chromatography-photodiode array/mass spectrometric/evaporative light scattering detection for quality control of black cohosh products. J Chromatogr A.

[B33] Ye M, Han J, Chen HB, Zheng JH, Guo DA (2007). Analysis of phenolic compounds in rhubarbs using liquid chromatography coupled with electrospray ionization mass spectrometry. J Am Soc Mass Spectrom.

[B34] Li YH, Jiang SY, Guan YL, Liu X, Zhou Y, Li LM, Huang SX, Sun HD, Peng SL, Zhou Y (2006). Quantitative determination of the chemical profile of the plant material "Qiang-huo" by LC-ESI-MS-MS. Chromatographia.

[B35] Wang CH (2003). GC-MS analysis of aconitine in biological tests. Zhongguo Fayixue Zazhi.

[B36] Sun XM, Dai DM, Chang XL, Yang XL (2007). Simultaneous determination of camphor, menthol, synthetic borneol and methyl salicylate in Shexiang Zhuanggu Plaster by GC. Zhongchengyao.

[B37] Yu K, Gong YF, Lin ZY, Cheng YY (2007). Quantitative analysis and chromatographic fingerprinting for the quality evaluation of Scutellaria baicalensis Georgi using capillary electrophoresis. J Pharmaceut Biomed.

[B38] Tong JY, Li W, Pan CM (2007). TLC identification and total flavonoids determination in herb sarcandra from different producing areas. Zhongyao Xinyao Yu Linchuang Yaoli.

[B39] Liu L, Ao L, Song J (2007). Determination of daphnetin in girald daphne bark fumigant by TCL-UV spectrophotometry. Zhongguo Yaofang.

[B40] Li YG, Zhang F, Wang ZT, Hu ZB (2004). Identification and chemical profiling of monacolins in red yeast rice using high-performance liquid chromatography with photodiode array detector and mass spectrometry. J Pharmaceut Biomed.

[B41] Yan SK, Xin WF, Luo GA, Wang YM, Chen YY (2005). Simultaneous determination of five groups of componentsin Qingkailing injection by high performance liquidchromatography with photo diode array detectorand evaporative light scattering detector. Sepu.

[B42] Guo FQ, Li A, Huang LF, Liang YZ, Chen BM (2006). Identification and determination of nucleosides in Cordyceps sinensis and its substitutes by high performance liquid chromatography with mass spectrometric detection. J Pharmaceut Biomed.

[B43] Ohtakea N, Nakaib Y, Yamamotoa M, Sakakibarab I, Takedaa S, Amagayab S, Aburadac M (2004). Review Separation and isolation methods for analysis of the active principles of Sho-saiko-to (SST) oriental medicine. J Chromatogr B.

[B44] Li XR, Liang YZ, Guo FQ (2006). Analysis of volatile oil in Rhizoma Ligustici Chuanxiong-Radix Paeoniae Rubra by gas chromatography-mass spectrometry and chemometric Resolution. Acta Pharmacologica Sinica.

[B45] Gong F, Liang YZ, Xu QS, Chau FT (2001). Gas chromatography-mass spectrometry and chemometric resolution applied to the determination of essential oils in *Cortex Cinnamomi*. J Chromatogr A.

[B46] Guo FQ, Liang YZ, Xu CJ, Huang LF (2003). Determination of the volatile chemical constituents of Notoptergium incium by gas chromatography-mass spectrometry and iterative or non-iterative chemometrics resolution methods. J Chromatogr A.

[B47] Guo FQ, Liang YZ, Xu CJ, Li XN, Huang LF (2004). Analyzing of the volatile chemical constituents in *Artemisia capillaris herba *by GC-MS and correlative chemometric resolution methods. J Pharmaceut Biomed.

[B48] Zhao CX, Li XN, Liang YZ, Fang HZ, Huang LF, Guo FQ (2006). Comparative analysis of chemical components of essential oils from different samples of Rhododendron with the help of chemometrics methods. Chemom Intell Lab Syst.

[B49] Zeng ZD, Liang YZ, Wang YL, Li XR, Liang LM, Xu QS, Zhao CX, Li BY, Chau FT (2006). Alternative moving window factor analysis for comparison analysis between complex chromatographic data. J Chromatogr A.

[B50] Gong F, Fung YS, Liang YZ (2004). Determination of volatile components in Ginger using gas chromatography-mass spectrometry with resolution improved by data processing techniques. J Agric Food Chem.

[B51] Gong F, Liang YZ, Cui H, Chau FT, Chan BTP (2001). Determination of volatile components in peptic powder by gas chromatography-mass spectrometry and chemometric resolution. J Chromatogr A.

[B52] Li XR, Lan ZG, Liang YZ (2007). Analysis of volatile chemical components of Radix Paeoniae Rubra by gas chromatography mass spectrometry and chemometric resolution. Zhongnan Gongye Daxue Xuebao.

[B53] Li BY, Liang YZ, Du YP, Xu CJ, Li XN, Song YQ, Cui H (2003). Resolution and identification of the acidic fraction of a petroleum ether extract of *Radix Rehmanniae Preparata *by an evolving chemometric approach. Chromatographia.

[B54] Li BY, Liang YZ, Xie PS, Yu RQ (2003). Spectral correlative chromatography and its application to the analysis of characteristic chemical fingerprints of traditional Chinese medicines. Fenxi Huaxue.

[B55] Qi YP, Sun SL, Wu YT, Li TH, Mi HM, Chai YF (2002). Application of heuristic evolving latent projection to the determination of loureirin B in resina draconis extracted by different methods. Fenxi Huaxue.

[B56] Zeng Z, Cheng YY, Shen GF (2003). A computational method for quantitative determining the active component of chinese medicine integrating PCA with GRAFA. Acta Chimica Sinica.

[B57] Makris DP, Kallithraka S, Mamalos A Differentiation of young red wines based on cultivar and geographical origin with application of chemometrics of principal polyphenolic constituents. Talanta.

[B58] Hu Y, Liang YZ, Li BoY, Li XN (2004). Multicomponent spectral correlative chromatography applied to complex herbal medicines. J Agric Food Chem.

[B59] Hu Y, Liang YZ, Li Bo Y, Xu CJ, Zeng ZD (2003). Multicomponent spectral correlative chromatography applied to analysis of chemical fingerprints of traditional Chinese medicines. Acta Chim Sinica.

[B60] Huang LF, Li BY, Liang YZ, Guo FQ, Wang YL (2004). Application of combined approach to analyze the constituents of essential oil from Dong quai. Anal Bioanal Chem.

[B61] Li BY, Hu Y, Liang YZ, Huang LF, Xu CJ, Xie PS (2004). Spectral correlative chromatography and its application to analysis of chromatographic fingerprints of herbal medicines. J Sep Sci.

[B62] Wang YL, Liang YZ, Hu Y, Li BY, Zeng ZD, Xu SP (2006). Comparison studies of constituents between dangguibuxuetang and its single medicines via hyphenated chromatography and multicomponent spectral correlative chromatography. Gaodeng Xuexiao Huaxue Xuebao.

[B63] Wang YL, Liang YZ, Hu Y, Li BY, Zeng ZD, He YK (2006). Study on absorption of Angelica sinensis in rabbit plasma by HPLC-DAD-MS and multicomponent spectral correlative chromatography. Chemom Intell Lab Syst.

[B64] Zeng YX, Zhao CX, Liang YZ, Yang H, Fang HZ, Yi LZ, Zeng ZD (2007). Comparative analysis of volatile components from *Clematis *species growing in China. Anal Chim Acta.

[B65] Yi LZ, Liang YZ, Zeng ZD, Yuan DL, Wang P (2006). AMWFA method applied to comparative analysis of two-dimensional data with overlapped peaks. Gaodeng Xuexiao Huaxue Xuebao.

[B66] Yi LZ, Liang YZ, Zeng ZD, Wang P, Yuan DL (2006). Comparative Analysis of Volatile Constituents in Chenpi of Different Original Plants by GC-MS and AMWFA. Gaodeng Xuexiao Huaxue Xuebao.

[B67] Cao YH, Wang LC, Yu XJ, Ye JN (2006). Development of the chromatographic fingerprint of herbal preparations Shuang-Huang-Lian oral liquid. J Pharmaceut Biomed.

[B68] Jin W, Ge RL, Wei QJ, Bao TY, Shi HM, Tu PF (2006). Short communication Development of high-performance liquid chromatographic fingerprint for the quality control of *Rheum tanguticum *Maxim. ex Balf. J Chromatogr A.

[B69] Fan XH, Wang Y, Cheng YY (2006). LC/MS fingerprinting of *Shenmai injection*: A novel approach to quality control of herbal medicines. J Pharmaceut Biomed.

[B70] Ruan GH, Li GK (2007). The study on the chromatographic fingerprint of *Fructus xanthii *by microwave assisted extraction coupled with GC-MS. J Chromatogr B.

[B71] Yan SK, Xin WF, Luo GA, Wang YM, Cheng YY (2005). An approach to develop two-dimensional fingerprint for the quality control of Qingkailing injection by high-performance liquid chromatography with diode array detection. J Chromatogr A.

[B72] Gu M, Ouyang F, Su ZG (2004). Comparison of high-speed counter-current chromatography and high-performance liquid chromatography on fingerprinting of Chinese traditional medicine. J Chromatogr A.

[B73] Gu M, Zhang SF, Su ZG, Chen Y, Ouyanga F (2004). Fingerprinting of Salvia miltiorrhiza Bunge by non-aqueous capillary electrophoresis compared with high-speed counter-current chromatography. J Chromatogr A.

[B74] Ma LJ, Zhang XZ, Zhang HP, Gan YR (2007). Development of a fingerprint of *Salvia miltiorrhiza Bunge *by high-performance liquid chromatography with a coulometric electrode array system. J Chromatogr B.

[B75] Li YP, Hu Z, He LC (2007). An approach to develop binary chromatographic fingerprints of the total alkaloids from Caulophyllum robustum by high performance liquid chromatography/diode array detector and gas chromatography/mass spectrometry. J Pharmaceut Biomed.

[B76] Fan XH, Cheng YY, Ye ZL, Lin RC, Qian ZZ (2006). Multiple chromatographic fingerprinting and its application to the quality control of herbal medicines. Anal Chim Acta.

[B77] Gu M, Su ZG, Ouyang F (2006). Fingerprinting of salvia miltiorrhiza bunge by thin-layer chromatography scan compared with high speed countercurrent chromatography. J Liquid Chromatogr R T.

[B78] Liu HX, Sun SQ, Lv GH, Chan KKC (2006). Study on *Angelica *and its different extracts by Fourier transform infrared spectroscopy and two-dimensional correlation IR spectroscopy. Spectrochim Acta A.

[B79] Zou HB, Yang GS, Qin ZR, Jiang WQ, Du AQ, Aboul-Enein HY (2005). Progress in quality control of herbal medicine with IR fingerprint spectra. Anal Lett.

[B80] Li ZX, Yuan CL, Nie F (2004). Application of chemical oscillation technique in herb's study. Zhongguo Jichu Kexue.

[B81] Zhang TM, Liang YZ, Yuan B, Ding F, Zhang YP, Wei MQ, Chen X (2007). Testing methods, conditions and factors of electrochemistry fingerprints of herbal medicines. Kexue Tongbao.

[B82] Zhang TM, Liang YZ, Yuan B, Ding F, Zhang YP, Chen WY (2007). Principles, features and applications of electrochemistry fingerprints of herbal medicines. Kexue Tongbao.

[B83] Yang JH, Hu EP, Guo LH, Li H (2006). Methodology of XRD second derivative fingerprint for traditional Chinese medicine. Nat Prod Res Dev.

[B84] Ran XR, Yang HH, Liang QL, Chen J, Wang YM, Luo GA, Li P, Li KM, Chen YW (2007). Difference analysis of mass spectra and its application to research of Chinese multiherb remedy. Gaodeng Xuexiao Huaxue Yanjiu.

[B85] Gong F, Wang BT, Chau FT, Liang YZ (2005). HPLC Data preprocessing for chromatographic fingerprint of herbal medicine with chemometric approaches. Anal Lett.

[B86] Gong F, Liang YZ, Xie PS, Chau FT (2003). Information theory applied to chromatographic fingerprint of herbal medicine for quality control. J Chromatogr A.

[B87] Gong F, Wang BT, Liang YZ, Chau FT, Fung YS (2006). Variable selection for discriminating herbal medicines with chromatographic fingerprints. Anal Chim Acta.

[B88] Xu CJ, Liang YZ, Chau FT, Heyden YV (2006). Pretreatments of chromatographic fingerprints for quality control of herbal medicines. J Chromatogr A.

[B89] Yang Y, Zhu XF (2007). Baseline spliting of traditional Chinese medicine fingerprint based on the second-generation wavelet transform. Huanan Ligong Daxue (Ziran Kexue Ban).

[B90] Fang KT, Liang YZ, Yin XL, Chan K, Lu GH (2006). Critical value determination on similarity of fingerprints. Chemom Intell Lab Syst.

[B91] Fan XH, Ye ZL, Cheng YY (2006). A computational method based on information fusion for evaluating the similarity of multiple chromatographic fingerprints of TCM. Gaodeng Xuexiao Huaxue Yanjiu.

[B92] Liu YS, Cao M, Wang YM, Luo GA (2006). Similarity system theory to evaluate the similarity of high performance liquid chromatography fingerprint of traditional Chinese medicine quantitatively. Fenxi Huaxue.

[B93] Wang K, Du K, Li H (2007). The similarity algorithm study on fingerprint of traditional Chinese medicine based on relative entropy. Comp Appl Chem.

[B94] Lu GH, Chan K, Liang YZ, Leung K, Chan CL, Jiang ZH, Zhao ZZ (2005). Development of high-performance liquid chromatographic fingerprints for distinguishing Chinese Angelica from related umbelliferae herbs. J Chromatogr A.

[B95] Zeng ZD, Liang YZ, Zhang T, Chau FT, Wang YL (2006). Orthogonal projection (OP) technique applied to pattern recognition of fingerprints of the herbal medicine houttuynia cordata Thunb. and its final injection products. Anal Bioanal Chem.

[B96] Yi LZ, Yuan DL, Liang YZ, Xie PS, Zhao Y (2007). Quality control and discrimination of Pericarpium Citri Reticulatae and Pericarpium Citri Reticulatae Viride based on high-performance liquid chromatographic fingerprints and multivariate statistical analysis. Anal Chim Acta.

[B97] Liu HX, Sun SQ, Lv GH, Liang XY (2006). Discrimination of extracted lipophilic constituents of Angelica with multi-steps infrared macro-fingerprint method. Vib Spectrosc.

[B98] Chen S, Zeng ZD, Liang YZ, Du YP, Yi LZ (2006). Realization and application of fingerprint database system of Herba houttuyniae injection. Comp App Chem.

[B99] Liu XH, Yu S, Cai BC, Qian JD (2005). Comprehensive database of TCM fingerprint. Zhongchengyao.

[B100] Wang YL, Liang YZ, Chen BM, He YK, Li BY, Hu QN (2005). LC-DAD-APCI-MS-based screening and analysis of the absorption and metabolite components in plasma from a rabbit administered an oral solution of danggui. Anal Bioanal Chem.

[B101] Lu HM, Liang YZ, Qian P (2005). Profile-effect on quality control of Houttuynia cordata injection. Acta Pharmaceutica Sinica.

[B102] Lu HM, Liang YZ, Yi LZ, Wu XJ (2006). Anti-inflammatory effect of *Houttuynia cordata *injection. J Ethnopharmacol.

[B103] Wang P, Liang YZ, Zhou N, Chen BM, Yi LZ, Yu Y, Yi ZB (2007). Screening and analysis of the multiple absorbed bioactive components and metabolites of Dangguibuxue decoction by the metabolic fingerprinting technique and liquid chromatography/diode-array detection mass spectrometry. Rapid Commun Mass Sp.

[B104] Yu Y, Yi ZB, Liang YZ (2007). Validate antibacterial mode and find main bioactive components of traditional Chinese medicine Aquilegia oxysepala. Bioorg Med Chem Lett.

[B105] Liang XM, Xu Q (2003). Strategy and methodology for analysis of active component fingerprint of traditional Chinese medicines. Zhongguo Tianran Yaowu.

